# Treatment and Cure of Nasal Catarrh

**Published:** 1882-06

**Authors:** 


### TREATMENT AND CURE OF NASAL CATARRH.

Dr. N. Ffealliott, writing to the *British Medical Journal*, states that coryza or nasal catarrh may be cured in a few hours if taken at the onset, or at most twelve hours afterward, by the inhalation of a spray of sulphate of quinine. The solution used is made by dissolving four grains of quinine in an ounce of water, with just sufficient dilute sulphuric acid to dissolve it, and scenting with any agreeable perfume. The solution is injected up the nostrils in the form of spray with an ordinary hand-ball spray producer in such a way that the quinine can be tasted at the back of the mouth. This is done at every hour or oftener, according to the urgency of the symptoms. He states that this remedy has been tried with success in hay fever, and that if nasal catarrh is of parasitic origin, as he strongly suspects, the action of quinine (as an antiseptic?) is at once apparent. It might be added that, even supposing catarrh to be the result of sudden change of temperature, the action of quinine in contracting the superficial capillaries would be quite as obvious. It is somewhat surprising that this property of quinine does not appear to have been tried for chilblains in the itching state, when the capillary vessels are dilated.

